# An Anisotropic Equivalent Thermal Model for Shield Differential Through-Silicon Vias

**DOI:** 10.3390/mi12101223

**Published:** 2021-10-07

**Authors:** Guangbao Shan, Guoliang Li, Dongdong Chen, Zifeng Yang, Di Li, Yintang Yang

**Affiliations:** School of Microelectronics, Xidian University, Xi’an 710071, China; gbshan@xidian.edu.cn (G.S.); guoliangli@stu.xidian.edu.cn (G.L.); yangzifeng99@gmail.com (Z.Y.); lidi2004@126.com (D.L.)

**Keywords:** equivalent model, through-silicon via, thermal analysis, thermal conductivity

## Abstract

An accurate equivalent thermal model is proposed to calculate the equivalent thermal conductivity (ETC) of shield differential through-silicon via (SDTSV). The mathematical expressions of ETC in both horizontal and vertical directions are deduced by considering the anisotropy of SDTSV. The accuracy of the proposed model is verified by the finite element method (FEM), and the average errors of temperature along the X-axis, Y-axis, diagonal line, and vertical directions are 1.37%, 3.42%, 1.76%, and 0.40%, respectively. Compared with COMSOL, the proposed model greatly improves the computational efficiency. Moreover, the effects of different parameters on the thermal distribution of SDTSV are also investigated. The thermal conductivity is decreased with the increase in thickness of SiO_2_. With the increase in pitch, the maximum temperature of SDTSV increases very slowly when β = 0° , and decreases very slowly when β = 90°. The proposed model can be used to accurately and quickly describe the thermal distribution of SDTSV, which has a great prospect in the design of 3D IC.

## 1. Introduction

Through-silicon via (TSV) is a key structure of 3D integrated circuits (IC) [1,2,3,4,5]. TSV-based 3D ICs have a shorter interconnect length, higher integration density, faster data communication, and lower power consumption [6,7,8,9,10]. Due to the excellent anti-noise characteristic during the transmission of different signals, shielded differential through-silicon vias (SDTSV) have wide prospects in 3D ICs [11]. However, with the increasing integration level of 3D ICs, the thermal effect obviously influences its performance, and has become an inevitable and urgent challenge [12,13,14]. Therefore, it is important to investigate the thermal effect on 3D ICs and establish an accurate thermal model of SDTSV for the design of 3D ICs.

In the last decade, the thermal models of TSV, coaxial TSV, and TSV arrays have been systematically investigated and established [15,16]. Meanwhile, the thermal models of 3D ICs have also been studied [17,18,19]. The thermal models in 3D ICs mainly include 1D networks, ETC models, the finite element method (FEM), and so on. Xiao et al. [20] established a fast and accurate equivalent thermal model for TSV. In this model, several parameters, including pitch, the thickness of SiO_2,_ and the radius of TSV, have been considered, and the accuracy of the proposed model has been verified by FEM. Hu et al. [19] proposed a thermal model of a high-power 3D-integrated RF module based on a TSV interposer, and the thermal model was verified by FEM. In addition, Chieh et al. [21] developed the equivalent thermal model of a TSV cell, and the maximum error was within 15%. Min et al. [22] proposed a 3D-equivalent thermal circuit model for coaxial TSV by equivalent thermal circuit, and the results showed that the proposed model worked well with FEM at transient temperatures. Feng et al. [23] proposed an equivalent thermal model for 2.5D packages, and the calculation time was reduced from 15 min to 23 s. Lau et al. [24] also investigated the thermal performance of three-layer-stacked chips using the computational fluid dynamics analysis method, and the empirical formula of effective thermal conductivity was proposed. Heng [25] proposed an equivalent model considering TSV, bump, and metallic trace, which could effectively improve the calculation efficiency of 3D system in package (SiP). Chen et al. [26] established an equivalent thermal resistance model for TSV, and the thermal performance of a 3D stacked-die package with TSV was quickly estimated by the proposed model. Pi et al. [17] established a fast and implementable full-chip scale numerical simulation method for thermal management of 3D ICs. In addition, the temperature difference is below 7.5%, and the grid number is reduced by 77%. Han et al. [27] proposed a thermal resistance network model based on 3D structures, and the proposed model could describe the temperature distribution of 3D structures. Konstantin O et al. [28] proposed an equivalent thermal model by using a quasi-3D approach, and the computational difficulties, processor time, and RAM volume were significantly reduced, while the simulation error of the maximal temperature was less than 20%. Wu et al. [29] proposed a thermal model using Laplace’s equation and the thermal resistance network, which could accurately predict the temperature of a heat source on the chip in these models; however, the 1D network was a simple model, and its accuracy was low. The accuracy of FEM is high, and generally used to verify the accuracy of the model, but it is complex and time consuming. TSV is treated as an equivalent thermal resistance in the equivalent model, which has high accuracy and calculation efficiency. Therefore, the ETC model can be used to describe the thermal distribution of SDTSV.

In recent years, SDTSV and derivative structures have been investigated by several researchers [30,31,32]. Lu et al. [11] proposed an equivalent circuit model of SDTSV in 3D ICs, and the proposed model worked well with the full-wave extraction method when the work frequency was up to 100 GHz. Fu et al. [30] investigated the equivalent circuit model of shielded differential annular through-silicon via, and analyzed its thermomechanical stress. Zhao et al. [31] proposed a novel differential TSV structure, and the accuracy of the circuit model was validated when the work frequency was up to 100 GHz. Liao et al. [32] proposed an equivalent electrical model for shielded-pair through-silicon vias, and the accuracy of the proposed model was verified. However, the thermal distribution of SDTSV is rarely investigated, whilst SDTSV plays a critical part in 3D ICs; therefore, it is necessary to establish an accurate ETC model for SDTSV to study its heat transfer performance. The equivalent thermal resistance models of typical and coaxial TSVs have been established in [11,22]. The thermal resistances in x and y directions are assumed to be the as the same in these models. However, the thermal resistances of SDTSV in different directions vary. In addition, SDTSV is composed of more oxide layers than common TSV and coaxial TSV. With the increase in oxide layers, finer grids are needed in the FEM, which can greatly increase the calculation burden and run-time; therefore, it is important to establish an accurate ETC model for SDTSV in order to quickly describe its heat distribution in both horizontal and vertical directions.

In this paper, an accurate and fast ETC model is established for SDTSV. The mathematical equations for the ETC of SDTSV are deduced in Section 2. The accuracy of the proposed ETC model is verified by FEM in Section 3, and the effects of different parameters on the thermal distribution of SDTSV are investigated and analyzed. Section 4 concludes this paper.

## 2. Expressions for Equivalent Thermal Conductivities of SDTSV

**Figure 1:**
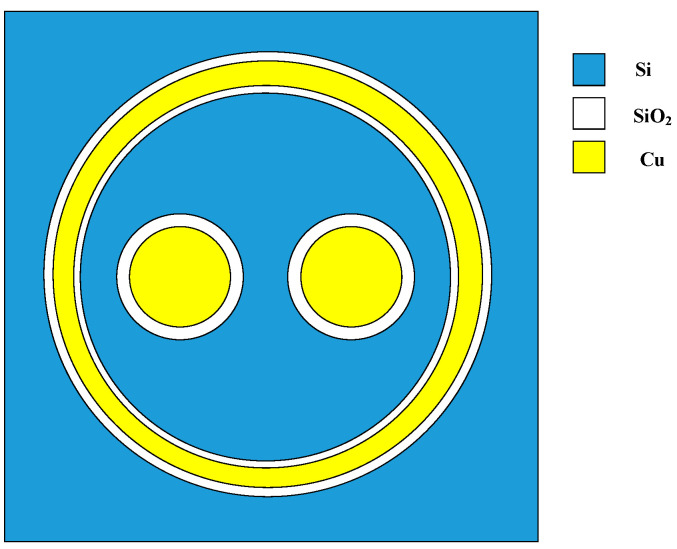
Shield differential through-silicon via.

The particular structure of SDTSV is shown in Figure 1 [11,30]. In this SDTSV, the differential signals are transmitted by the inner two TSVs, and the function of the outer shell is shielding the external signal interference. The inner two TSVs are filled with Cu, and surrounded by the oxide insulation layer. The main structure parameters of SDTSV are shown in Table 1. Compared with TSV and coaxial TSV, the thermal distribution of SDTSV in horizontal directions may be affected by the number and location of inner TSVs; thus, the ETCs of SDTSV in horizontal and vertical directions are deduced to describe the thermal distribution of SDTSV. Subsequently, an accurate ETC model is established for SDTSV with an area of a × b. Three assumptions for the ETC model of SDTSV can be described: (1) since the horizontal temperature gradient is relatively large and the most of thermal flow comes from the outside, the heat generated by SDTSV can be ignored; (2) due to the isotropy of materials in different layers, the thermal resistances among different layers can be ignored; and (3) since the heat flow is assumed to be straight in the SDTSV, the detour of heat flow close to the other layer can be considered negligible.

When the heat flow Q in any direction is applied to SDTSV, it is divided into vertical and horizontal components, and the ETC model in the vertical direction is deduced first. In this model, the heat flow along the copper (Cu), silicon dioxide (SiO_2_), and silicon (Si) can be assumed as the parallel relationship, and the thermal resistance $R_{\mathrm{eff}\_\mathrm{vertical}}$ in the vertical direction for SDTSV cell can be calculated by

(1) $$1/R_{eff\_vertical}=1/R_{Si\text{\_}\mathrm{sub}}+1/R_{SiO_{2}\_1}+1/R_{Cu\_outside}+1/R_{SiO_{2}\_2}+1/R_{Si\_inner}+1/R_{SiO_{2}\_3}+1/R_{Cu\_inner}$$

where $R_{Si\_sub}$ is the thermal resistance of the Si substrate; $R_{SiO_{2}\_1}$ is the thermal resistance of outer oxide insulation layer of shielding shell; $R_{Cu\_outside}$ is the thermal resistance of metal layer of shielding shell; $R_{SiO_{2}\_2}$ is the thermal resistance of inner oxide insulation layer of shielding shell; $R_{Si\_inner\;}$ is the thermal resistance of inner Si; $R_{SiO_{2}\_3}$ is the thermal resistance of oxide insulation layer of inner signal TSV; and $R_{Cu\_inner}$ is the thermal resistance of the copper core of the inner signal TSV. The ETC $K_{eff\_vertical}$ of SDTSV in vertical direction can be obtained by

(2) $$\begin{array}{cc} K_{eff\_vertical} & =[K_{Si}\left(ab-\pi r_{1}^{2}\right)+K_{SiO_{2}}\pi \left(r_{1}^{2}-r_{2}^{2}\right)+K_{Cu}\pi \left(r_{2}^{2}-r_{3}^{2}\right)+K_{SiO_{2}}\pi \left(r_{3}^{2}-r_{4}^{2}\right)\\ & +K_{Si}\pi \left(r_{4}^{2}-2r_{5}^{2}\right)+K_{SiO_{2}}2\pi \left(r_{5}^{2}-r_{6}^{2}\right)+K_{Cu}2\pi r_{6}^{2}]/\left(a\cdot b\right) \end{array}$$

According to Equation (2), $K_{eff\_vertical}$ is mainly related to the area ratio of Cu, SiO_2,_ and Si in the SDTSV cell.

**Figure 2:**
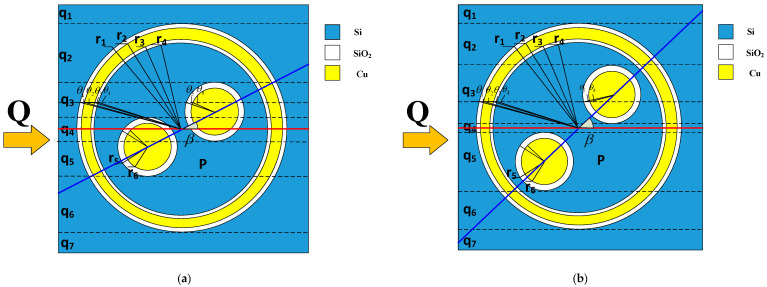
Shield differential through-silicon via in (**a**) case 1; (**b**) case 2.

The horizontal section of SDTSV is shown in Figure 2, and the thermal conductivities of SDTSV in different directions are distinct. The proposed model is established in the Cartesian coordinate system. The direction of the heat flow is fixed, and the SDTSV is rotated to simulate the heat flow coming from different directions and passing through the SDTSV unit. The blue line connects the centers of two inner metal holes, and the red line is parallel to the X-axis. The angle between the blue line and the red line is the rotation angle β. Based on the relationship of the rotation angle β, the pitch of inner metal holes p, and the oxide radius of the TSV r_5_, the thermal conductivity of SDTSV in the horizontal direction can be divided into two cases, as shown in Figure 2. In Figure 2a, the heat flow can be roughly split into seven regions (q1, q2, q3, q4, q5, q6, and q7) when $sin\beta <2\times r_{5}/p$. The thermal resistance $R_{horizontal}$ can be calculated by

(3) $$1/R_{horizontal}=1/R_{q1}+1/R_{q2}+1/R_{q3}+1/R_{q4}+1/R_{q5}+1/R_{q6}+1/R_{q7}$$

The materials in q1 and q7 are Si, and the thermal resistances in q1 and q7 can be calculated by Equation (5). Meanwhile, the thermal resistance in q2 and q6 can be obtained by Equation (6), and the thermal resistance in q3 and q5 can be obtained by Equation (7). The thermal conductivity in q4 can be obtained by Equation (8). Accordingly, the ETC of SDTSV can be calculated by Equation (4) when $sin\beta <2\times r_{5}/p$.

(4) $$K_{case1}=\frac{a}{2\times b\times h}\left(1/R_{q1}+1/R_{q2}+1/R_{q3}+1/R_{q4}+1/R_{q5}+1/R_{q6}+1/R_{q7}\right)$$

(5) $$R_{q1}=R_{q7}=\frac{K_{Si}\times (b-2r_{1})\times h}{2a}$$

(6) $$\begin{array}{cc} R_{q2} & =R_{q6}=\frac{1}{2}\int_{p\sin\beta /2+r_{5}}^{r_{1}}[\frac{\left(b-2r_{1}\cos\theta_{1}\right)}{K_{Si}hdy}+2\frac{\left(r_{1}\cos\theta_{1}-r_{2}\cos\theta_{2}\right)}{K_{SiO_{2}}hdy}+2\frac{\left(r_{2}\cos\theta_{2}-r_{3}\cos\theta_{3}\right)}{K_{Cu}hdy}\\ & +2\frac{\left(r_{3}\cos\theta_{3}-r_{4}\cos\theta_{4}\right)}{K_{SiO_{2}}hdy}+2\frac{r_{4}\cos\theta_{4}}{K_{Si}hdy}] \end{array}$$

(7) $$\begin{array}{cc} R_{q3} & =\int_{r_{5}-p\cdot \sin\beta /2}^{p\cdot \sin\beta /2+r_{5}}[\frac{b-2r_{1}\cos\theta_{1}+2r_{4}\cos\theta_{4}-2r_{5}\cdot \cos\theta_{5}}{K_{Si}hdy}+\frac{2r_{1}\cos\theta_{1}-2r_{2}\cos\theta_{2}}{K_{SiO_{2}}hdy}+\frac{2r_{3}\cos\theta_{3}-2r_{4}\cos\theta_{4}}{K_{SiO_{2}}hdy}\\ & +\frac{2r_{5}\cos\theta_{5\_metal1}-2r_{6}\cos\theta_{6\_metal1}}{K_{SiO_{2}}hdy}+\frac{2r_{2}\cos\theta_{2}-2r_{3}\cos\theta_{3}+2r_{6}\cos\theta_{6\_metal1}}{K_{Cu}hdy}] \end{array}$$

(8) $$\begin{array}{cc} R_{q4} & =\int_{r_{5}-p\cdot \sin\beta /2}^{p\cdot \sin\beta /2-r_{5}}[\frac{b-2r_{1}\cos\theta_{1}+2r_{4}\cos\theta_{4}-2r_{5}\cdot \cos\theta_{5\_metal1}-2r_{5}\cdot \cos\theta_{5\_metal2}}{K_{Si}hdy}+\frac{2r_{1}\cos\theta_{1}-2r_{2}\cos\theta_{2}}{K_{SiO_{2}}hdy}+\frac{2r_{3}\cos\theta_{3}-2r_{4}\cos\theta_{4}}{K_{SiO_{2}}hdy}\\ & +\frac{2r_{5}\cos\theta_{5\_metal1}-2r_{6}\cos\theta_{6\_metal1}+2r_{5}\cos\theta_{5\_metal2}-2r_{6}\cos\theta_{6\_metal2}}{K_{SiO_{2}}hdy}+\frac{2r_{6}\cos\theta_{6\_metal1}+2r_{6}\cos\theta_{6\_metal2}}{K_{Cu}hdy}] \end{array}$$

(9) $$\begin{array}{cc} R_{q5} & =\int_{-r_{5}-p\cdot \sin\beta /2}^{r_{5}-p\cdot \sin\beta /2}[\frac{b-2r_{1}\cos\theta_{1}+2r_{4}\cos\theta_{4}-2r_{5}\cdot \cos\theta_{5}}{K_{Si}hdy}+\frac{2r_{1}\cos\theta_{1}-2r_{2}\cos\theta_{2}}{K_{SiO_{2}}hdy}+\frac{2r_{3}\cos\theta_{3}-2r_{4}\cos\theta_{4}}{K_{SiO_{2}}hdy}\\ & +\frac{2r_{5}\cos\theta_{5\_metal2}-2r_{6}\cos\theta_{6\_metal2}}{K_{SiO_{2}}hdy}+\frac{2r_{2}\cos\theta_{2}-2r_{3}\cos\theta_{3}+2r_{6}\cos\theta_{6\_metal2}}{K_{Cu}hdy}] \end{array}$$

(10) $$\theta_{n}=\arcsin\frac{c}{r_{n}}\left(n=1,2,3,4,5,6\right)$$

where $\theta_{n}$ are the angles between radius $r_{n}$ and the X-axis. From Equations (4)–(10), $K_{case1}$ is determined by the radius and the thermal conductivity of materials in different parts. $\theta_{5\_metal1}$ and $\theta_{5\_metal2}$ are the angles between radius $r_{5}$ and the X-axis of two inner metal holes, and $\theta_{6\_metal1}$ and $\theta_{6\_metal2}$ are the angles between radius $r_{6}$ and the X-axis of two inner metal holes. cis the distance from the center of the circle to the transversal line that is parallel to X-axis.

In the Figure 2b, the thermal resistance $R_{horizontal}$ can be roughly split into seven regions (q1, q2, q3, q4, q5, q6, and q7), where $sin\beta \geq 2\times r_{5}/p$. Similarly, the thermal resistance $R_{case2}$ can be calculated by

(11) $$1/R_{horizontal}=1/R_{q1}+1/R_{q2}+1/R_{q3}+1/R_{q4}+1/R_{q5}+1/R_{q6}+1/R_{q7}$$

Similarly, the ETC in case 2 $K_{case2}$ of SDTSV can be calculated by Equation (12).

(12) $$K_{case2}=\frac{b}{2\times a\times h}\left(\frac{1}{R_{q1}}+\frac{1}{R_{q2}}+\frac{1}{R_{q3}}+\frac{1}{R_{q4}}+\frac{1}{R_{q5}}+\frac{1}{R_{q6}}+\frac{1}{R_{q7}}\right)$$

(13) $$R_{q1}=R_{q7}=\frac{2K_{Si}\times (b-2r_{1})\times h}{a}$$

(14) $$\begin{array}{cc} R_{q2} & =R_{q6}=\frac{1}{2}\int_{p\cdot \sin\beta /2+r_{5}}^{r_{1}}[\frac{\left(b-2r_{1}\cos\theta_{1}\right)}{K_{Si}hdy}+2\frac{\left(r_{1}\cos\theta_{1}-r_{2}\cos\theta_{2}\right)}{K_{SiO_{2}}hdy}+2\frac{\left(r_{2}\cos\theta_{2}-r_{3}\cos\theta_{3}\right)}{K_{Cu}hdy}\\ & +2\frac{\left(r_{3}\cos\theta_{3}-r_{4}\cos\theta_{4}\right)}{K_{SiO_{2}}hdy}+2\frac{r_{4}\cos\theta_{4}}{K_{Si}hdy}] \end{array}$$

(15) $$\begin{array}{cc} R_{q3} & =\frac{1}{2}\int_{p\cdot \sin\beta /2-r_{5}}^{p\cdot \sin\beta /2+r_{5}}[\frac{\left(b-2r_{1}\cos\theta_{1}\right)}{K_{Si}hdy}+2\frac{\left(r_{1}\cos\theta_{1}-r_{2}\cos\theta_{2}\right)}{K_{SiO_{2}}hdy}+2\frac{\left(r_{2}\cos\theta_{2}-r_{3}\cos\theta_{3}\right)}{K_{Cu}hdy}\\ & +2\frac{\left(r_{3}\cos\theta_{3}-r_{4}\cos\theta_{4}\right)}{K_{SiO_{2}}hdy}+2\frac{\left(r_{4}\cos\theta_{4}-r_{5}\cos\theta_{5\_metal1}\right)}{K_{Si}hdy}+2\frac{\left(r_{5}\cos\theta_{5\_metal1}-r_{6}\cos\theta_{6\_metal1}\right)}{K_{SiO_{2}}hdy}+2\frac{r_{6}\cos\theta_{6\_metal1}}{K_{Cu}hdy}] \end{array}$$

(16) $$\begin{array}{cc} R_{q4} & =\int_{r_{5}-p\cdot \sin\beta /2}^{p\cdot \sin\beta /2-r_{5}}[\frac{\left(b-2r_{1}\cos\theta_{1}\right)}{K_{Si}hdy}+2\frac{\left(r_{1}\cos\theta_{1}-r_{2}\cos\theta_{2}\right)}{K_{SiO_{2}}hdy}+2\frac{\left(r_{2}\cos\theta_{2}-r_{3}\cos\theta_{3}\right)}{K_{Cu}hdy}\\ & +2\frac{\left(r_{3}\cos\theta_{3}-r_{4}\cos\theta_{4}\right)}{K_{SiO_{2}}hdy}+2\frac{r_{4}\cos\theta_{4}}{K_{Si}hdy}] \end{array}$$

(17) $$\begin{array}{cc} R_{q5} & =\frac{1}{2}\int_{-p\cdot \sin\beta /2-r_{5}}^{r_{5}-p\cdot \sin\beta /2}[\frac{\left(b-2r_{1}\cos\theta_{1}\right)}{K_{Si}hdy}+2\frac{\left(r_{1}\cos\theta_{1}-r_{2}\cos\theta_{2}\right)}{K_{SiO_{2}}hdy}+2\frac{\left(r_{2}\cos\theta_{2}-r_{3}\cos\theta_{3}\right)}{K_{Cu}hdy}\\ & +2\frac{\left(r_{3}\cos\theta_{3}-r_{4}\cos\theta_{4}\right)}{K_{SiO_{2}}hdy}+2\frac{\left(r_{4}\cos\theta_{4}-r_{5}\cos\theta_{5\_metal2}\right)}{K_{Si}hdy}+2\frac{\left(r_{5}\cos\theta_{5\_metal2}-r_{6}\cos\theta_{6\_metal2}\right)}{K_{SiO_{2}}hdy}+2\frac{r_{6}\cos\theta_{6\_metal2}}{K_{Cu}hdy}] \end{array}$$

Equations (11)–(17) show that $K_{case2}$ is determined by the radius, pitch between two inner signal TSVs, and the thermal conductivity of materials in different parts.

## 3. Model Validation and Discussion

The accuracy of the ETC model established in the previous section is verified by FEM, and the effects of the parameters on the thermal distribution are investigated. In addition, the error of the proposed model is analyzed.

### 3.1. Numerical Validation

**Figure 3:**
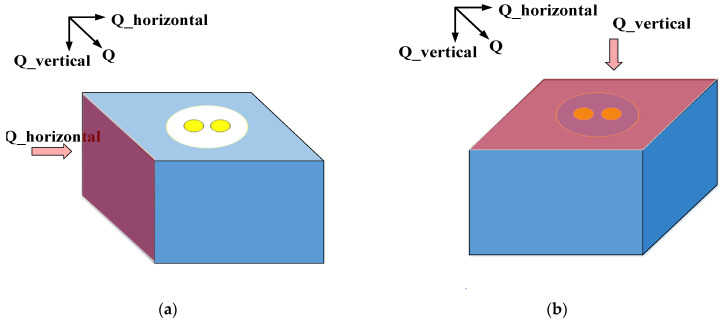
Stereogram of SDTSV for thermal conductivity verification in (**a**) horizontal direction and (**b**) vertical direction.

The accuracy of the proposed ETC model is verified by COMSOL. In this study, the numerical solutions of Equations (1)–(17) are solved by MATLAB, and the results are compared with those obtained by COMSOL. The parameters of FEM are shown in Table 2. In particular, the thickness of SiO_2_ is set to 0.2 μm, the rotation angles β are set to 0° (parallel to X-axis), 45° (diagonal line) and 90° (parallel to Y-axis), and the pitch of the inner metal hole is set from 25 μm to 90 μm, with a step size of 5 μm. The established SDTSV structure is shown in Figure 3. In this simulation, the heat flux Q (300 W/cm^2^) is imposed on the left [33,34]. The opposite side of the heat surface is set to the constant temperature, and the other surfaces have no heat exchange with the outside. The initial temperature of the SDTSV is set to 20℃ [26,35,36,37]. The experiments were performed using COMSOL 5.6 software with a 2.7 GHz quad-core CPU.

**Figure 4:**
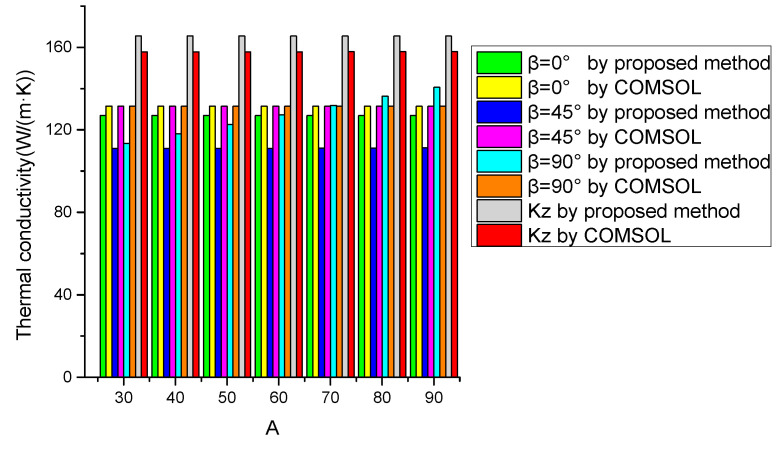
Comparison between the proposed model and COMSOL.

The comparison between the proposed model and COMSOL is shown in Figure 4. The thermal conductivities obtained by the proposed model have a good agreement with those obtained by COMSOL. When the rotation angle β is equal to 0°, 45°, and 90°, the maximum deviations between the two models are 6.91%, 10.51%, and 6.96%, respectively. The maximum temperature of SDTSV and run-time of the two models are shown in Table 3. The maximum deviations between the two models when the rotation angle β is equal to 0°, 45°, 90° are 1.37%, 3.42%, and 1.76%, respectively. The maximum deviations between the two models in a vertical direction are 0.40%, which implies that the proposed model can accurately describe the maximum temperature of SDTSV. In addition, the calculation efficiency of the proposed model is greatly improved compared with that of COMSOL.

**Figure 5:**
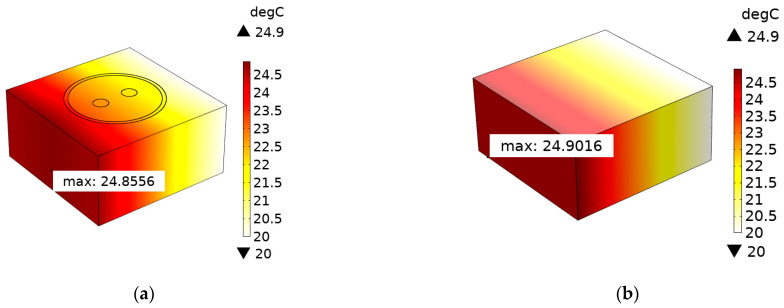
The 3D temperature distributions of SDTSV obtained by: (**a**) COMSOL and (**b**) the proposed model.

**Figure 6:**
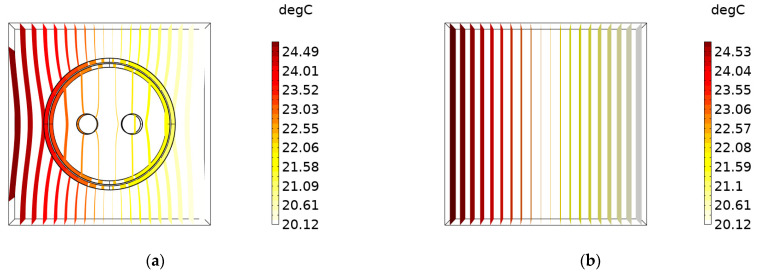
Isotherms of SDTSV obtained by: (**a**) COMSOL and (**b**) the proposed model.

The 3D temperature distributions of SDTSV obtained by the proposed model and COMSOL are shown in Figure 5. Obviously, the temperature distribution obtained by the proposed model is in accordance with that obtained by COMSOL, and the maximum error of the two models is 0.394 K, which also indicates that the proposed model can precisely simulate the thermal distribution of SDTSV. Isotherms of SDTSV obtained by the proposed model and COMSOL are shown in Figure 6. As shown in Figure 6a, the isotherm makes a detour in the interfaces of different martials, whereas the isotherm obtained by the proposed model is smooth. This is because SiO_2_ has a poorer heat-transfer performance than Cu and Si, which results in the detour of the isotherm. However, the SDTSV is treated as a lump model in the proposed model, which ignores the inside features, so the isotherm is smooth.

### 3.2. Effects of Different Parameters on the Temperature Distribution of SDTSV

The accuracy of the proposed model is verified above. Based on the proposed model, the effects of different parameters (the pitch and thickness of SiO_2_) on the thermal conductivity of SDTSV are investigated. In particular, the thickness of SiO_2_ is set from 0.1 μm to 0.6 μm, with a step size of 0.1 μm, and the pitch between the TSVs in SDTSV changes from 25 μm to 90 μm, with a step size of 5 μm. The ETCs of SDTSV in horizontal and vertical directions obtained by COMSOL are calculated by

(18) $$K_{m\_comsol}=q\frac{\Delta m}{\Delta S\times \Delta T},m=x,y,z$$

where Δm is the length of heat flow through the conductor, ΔS is the cross-sectional area of heat flow through the conductor, and ΔT is the temperature drop of heat flow through the conductor.

**Figure 7:**
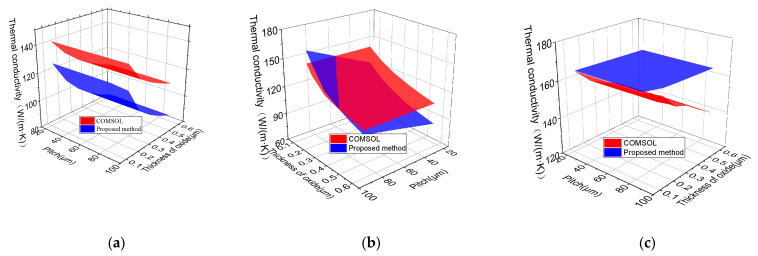
Effects of different parameters on equivalent thermal conductivity in (**a**) x-direction; (**b**) y-direction; (**c**) z-direction.

The effects of different parameters on ETC in x, y and z directions are shown in Figure 7. The red surface is the result obtained COMSOL, and the blue surface is the result of the proposed model. As shown in Figure 7a, the ETC obtained by COMSOL decreases as the SiO_2_ thickness increases, but it changes little with the increase in pitch. When the SiO_2_ thickness is below 0.3 μm, the maximum error of thermal conductivity is 10.39%. Since the thermal conductivity of SiO_2_ is much lower than that of Si and Cu, the ETC decreases with the increase in SiO_2_ thickness. In addition, simulation results show that the pitch of the inner metal has little influence on the thermal distribution of SDTSV; however, the variation position of the isotherm is affected by the pitch of inner metals.

As shown in Figure 7b, the ETC obtained by COMSOL decreases with the increase in SiO_2_ thickness, but it changes little with the increase in pitch. The ETC obtained by the proposed model increases with the increase in pitch, and it decreases with the increase in SiO_2_ thickness. In addition, the error of thermal conductivities between the proposed model and FEM increases with the increase in SiO_2_ thickness. This is because when the thickness of SiO_2_ is larger than 0.4 μm, the heat flow along the tangential direction cannot be ignored; however, this situation is not considered in the proposed model.

As shown in Figure 7c, the ETC obtained by FEM changes little with the increase in pitch, and it decreases with the increase in SiO_2_ thickness. The ETC obtained by the proposed model is in accordance with that obtained by FEM. When the thickness of SiO_2_ is less than 0.4 μm, the maximum error of thermal conductivity is 9.61%.

**Figure 8:**
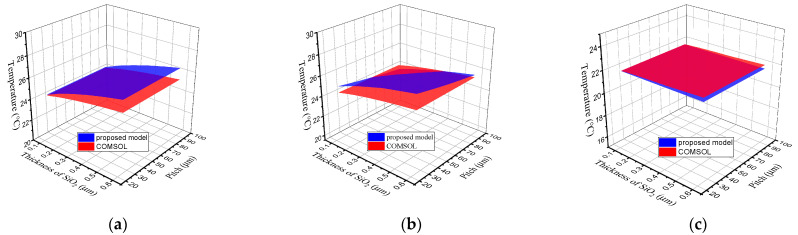
Effects of different parameters on the thermal distribution in (**a**) x-direction; (**b**) y-direction; and (**c**) z-direction.

The effects of different parameters on the thermal distributions of SDTSV in x, y, and z directions are shown in Figure 8. The variation trends in temperature are consistent with those of thermal conductivities in x, y, and z directions. In addition, the maximum errors of steady-state temperature are 5.53%, 4.21%, and 1.44%, respectively. As such, the proposed model is highly accurate.

### 3.3. Thermal Distribution of SDTSV Array

**Figure 9:**
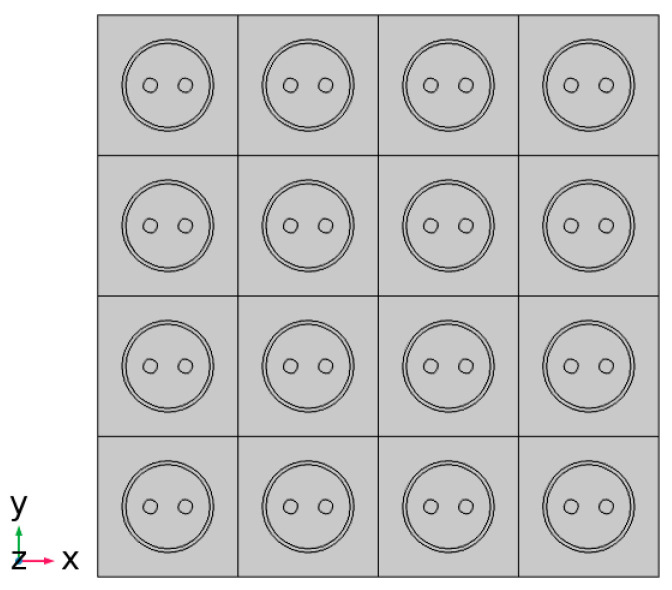
The 4 × 4 SDTSV array.

**Figure 10:**
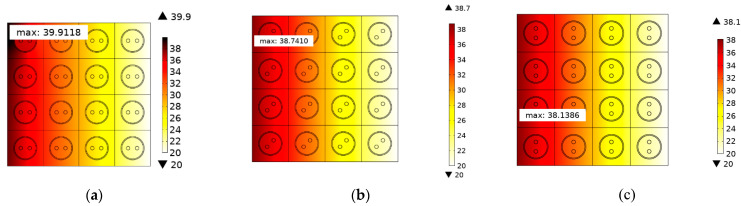
Temperature distribution of 4×4 SDTSV array obtained by COMSOL when the rotation is equal to (**a**) 0°; (**b**) 45°; and (**c**) 90°.

**Figure 11:**
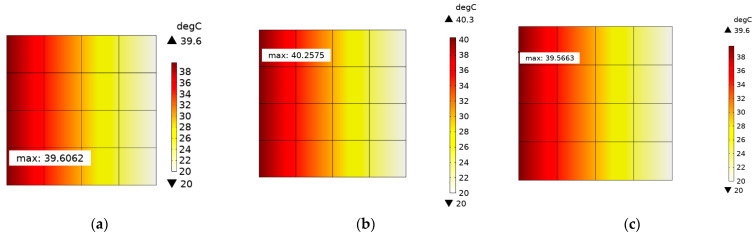
Temperature distribution of 4×4 SDTSV array obtained by the proposed model when the rotation is equal to (**a**) 0°; (**b**) 45°; and (**c**) 90°.

The thermal distributions of SDTSV array in x and y directions are further investigated in this section. A 4×4 SDTSV array is established, in which the pitch is 50 μm and the thickness of the SiO_2_ is 0.2 μm. The structure of the SDTSV array is shown in Figure 9. The heat flux Q (300 W/cm^2^) is imposed on the left. The opposite side of the heat surface is set to 20 °C, and the other surfaces have no heat exchange with the outside. The simulation results of the temperature distribution by FEM are shown in Figure 10a–c. Obviously, the temperature is decreased along the direction of heat conduction. In addition, the results of temperature distribution obtained by the proposed model are shown in Figure 11a–c. The errors of maximum temperature between COMSOL and the proposed model are 0.3056 K, 1.4277 K, and 1.5165 K when the rotation angle β is equal to 0°, 45°, and 90°, respectively. As such, the temperature distribution of the SDTSV array obtained by the proposed model has a good agreement with that obtained by FEM.

### 3.4. Discussion and Analysis

Based on the comparisons, the accuracy of the proposed model is verified by COMSOL. The maximum temperature error is lower than 1.5165 K, which is relatively small. In addition, compared with FEM, the calculation time of the proposed model is reduced from approximately 300 s to 1.9 s. However, the error of equivalent model is inevitable, which is its limitation. Compared with the finite element method (FEM), the equivalent model greatly improves the computational efficiency at the expense of certain accuracy. In the published studies, the maximum error of thermal conductivity in [21] is approximately 15%, and the temperature difference in [17] is below 7.5%. The simulation error of maximal temperature in [28] is less than 20%. The error of the proposed model is within 10% for thermal conductivity, and the maximum temperature error is 5.53%. As such, the proposed model is relatively accurate in comparison with other models. In addition, the heat flow is assumed to propagate along a straight line in the proposed model. However, due the heat flow propagating along the direction of small thermal resistance in practice, the detour phenomenon exists in the thermal propagation, which leads to the error in the proposed model. Moreover, compared with TSV and coaxial TSV, the structure of SDTSV is more complicated, which leads to the complex heat flow. In our future research, we will further study the characteristics of heat propagation in multi-layer media, so as to establish a more accurate equivalent thermal model.

According to the results obtained by COMSOL, the ETC in a vertical direction is larger than in a horizontal direction. This is because the Cu runs through the SDTSV along the vertical direction, and it is treated as the main channel of thermal dissipation. When β is equal to 0°, 45°, and 90°, the simulation results show that the thermal conductivities of the SDTSV cell are nearly equal. This is due to the radius of inner metal being very small, which has little effect on the thermal conductivity in horizontal directions. However, the maximum temperature of the SDTSV array obtained by COMSOL at β = 0° is higher than that at β = 90°, and the difference in maximum temperatures between β = 0° and β=90° is approximately 1.77 K in the 4×4 SDTSV array. This is because the thermal conductivities at β = 0°, 45°, and 90° are slightly different, and the difference in temperature is accumulated during thermal conduction along different directions; therefore, the difference in thermal dissipation in horizontal directions is magnified in the SDTSV array. In our future research, the effects of different parameters on the thermal distribution of SDTSV array will be studied, and the design and fabrication of SDTSV will be investigated to be applied in differential signal transmission of 3D ICs.

## 4. Conclusions

In this paper, an accurate equivalent thermal model is proposed to describe the thermal distribution of SDTSV, and the main conclusions can be summarized as follows:

(1) An accurate equivalent thermal model is established for SDTSV, which can describe the thermal distribution of SDTSV along an arbitrary angle. The mathematical expressions of ETC in horizontal and vertical directions are deduced;
(2) The accuracy of the proposed model is verified by COMSOL, and the average errors of temperature at β=0° , 45°, 90° and vertical directions are 1.37%, 3.42%, 1.76%, and 0.40%, respectively. In addition, the calculation time of the proposed model is reduced from approximately 300 s to 1.92 s;

(3) The effects of different parameters on the thermal distribution of SDTSV are investigated based on the proposed model. As the pitch increases, the maximum temperature of SDTSV increases very slowly when β = 0° , and decreases very slowly when β = 90°; however, the ETC decreases as the thickness of SiO_2_ increases.

## Figures and Tables

**Table 1:** Main structure parameters of SDTSV.

Symbol	Definition
$h_{TSV}$	the height of TSV
p	pitch between two inner signal TSVs
$t_{ox}$	oxide thickness
$r_{1}$	outer oxide radius of the shielding shell
$r_{2}$	outer metal radius of the shielding shell (=r1 − tox)
$r_{3}$	inner metal radius of the shielding shell
$r_{4}$	inner oxide radius of the shielding shell (=r3 − tox)
$r_{5}$	oxide radius of the TSV
$r_{6}$	metal radius of the TSV (=r5 − tox)
$K_{si}$	the thermal conductivity of Si
$K_{SiO_{2}}$	the thermal conductivity of SiO_2_
$K_{Cu}$	the thermal conductivity of Cu
a	the width of SDTSV cell
b	the length of SDTSV cell

**Table 2:** Model parameters.

Symbol	Definition	Value
$h_{TSV}$	the height of TSV	100 μm
$t_{ox}$	oxide thickness	0.2 μm
$r_{1}$	outer oxide radius of the shielding shell	10.2 μm
$r_{2}$	outer metal radius of the shielding shell(=r1−tox)	10 μm
$r_{3}$	nner metal radius of the shielding shell	60 μm
$r_{4}$	inner oxide radius of the shielding shell(=r3−tox)	59.8 μm
$r_{5}$	oxide radius of the TSV	65.2 μm
$r_{6}$	metal radius of the TSV (=r5−tox)	65 μm
$K_{si}$	the thermal conductivity of Si	150 K/(W·m)
$K_{SiO_{2}}$	the thermal conductivity of SiO_2_	1.38 K/(W·m)
$K_{Cu}$	the thermal conductivity of Cu	400 K/(W·m)
a	the width of SDTSV cell	200 μm
b	the length of SDTSV cell	200 μm

**Table 3:** Error and run-time of different models.

Model	Max. Temperature (°C)									Av. Error	Av. Time (s)
	Pitch (μm)	25	30	35	40	45	50	55	60	-	-
Proposed method	β=0°	24.83	24.73	24.63	24.54	24.45	24.36	24.28	24.19	1.37%	1.92
	β=45°	25.41	25.41	25.41	25.41	25.41	25.41	25.41	25.40	3.42%	1.89
	β=90°	25.40	25.29	25.18	25.08	24.99	24.89	24.80	24.71	1.76%	1.87
	Qz	21.81	21.81	21.81	21.81	21.81	21.81	21.81	21.81	0.40%	1.96
COMSOL	β=0°	24.56	24.56	24.56	24.56	24.56	24.57	24.57	24.57	-	297
	β=45°	24.57	24.57	24.57	24.57	24.57	24.57	24.57	24.57	-	287
	β=90°	24.57	24.57	24.57	24.57	24.57	24.57	24.57	24.57	-	293
	Qz	21.90	21.90	21.90	21.90	21.90	21.90	21.90	21.90	-	306
